# Process intensification of EB66® cell cultivations leads to high-yield yellow fever and Zika virus production

**DOI:** 10.1007/s00253-018-9275-z

**Published:** 2018-08-08

**Authors:** Alexander Nikolay, Arnaud Léon, Klaus Schwamborn, Yvonne Genzel, Udo Reichl

**Affiliations:** 1 Max Planck Institute for Dynamics of Complex Technical Systems, Bioprocess Engineering, Magdeburg, Sandtorstr. 1, 39106 Magdeburg, Germany; 2 Valneva SE, 6 rue Alain Bombard, 44800 Saint-Herblain, France; 3 Otto von Guericke University Magdeburg, Universitätsplatz 2, 39106 Magdeburg, Germany

**Keywords:** EB66^®^, Yellow fever virus, Zika virus, Flavivirus, Perfusion, Capacitance probe

## Abstract

A live-attenuated, human vaccine against mosquito-borne yellow fever virus has been available since the 1930s. The vaccine provides long-lasting immunity and consistent mass vaccination campaigns counter viral spread. However, traditional egg-based vaccine manufacturing requires about 12 months and vaccine supplies are chronically close to shortages. In particular, for urban outbreaks, vaccine demand can be covered rarely by global stockpiling. Thus, there is an urgent need for an improved vaccine production platform, ideally transferable to other flaviviruses including Zika virus. Here, we present a proof-of-concept study regarding cell culture-based yellow fever virus 17D (YFV) and wild-type Zika virus (ZIKV) production using duck embryo-derived EB66® cells. Based on comprehensive studies in shake flasks, 1-L bioreactor systems were operated with scalable hollow fiber-based tangential flow filtration (TFF) and alternating tangential flow filtration (ATF) perfusion systems for process intensification. EB66® cells grew in chemically defined medium to cell concentrations of 1.6 × 10^8^ cells/mL. Infection studies with EB66®-adapted virus led to maximum YFV titers of 7.3 × 10^8^ PFU/mL, which corresponds to about 10 million vaccine doses for the bioreactor harvest. For ZIKV, titers of 1.0 × 10^10^ PFU/mL were achieved. Processes were automated successfully using a capacitance probe to control perfusion rates based on on-line measured cell concentrations. The use of cryo-bags for direct inoculation of production bioreactors facilitates pre-culture preparation contributing to improved process robustness. In conclusion, this platform is a powerful option for next generation cell culture-based flavivirus vaccine manufacturing.

**Electronic supplementary material:**

The online version of this article (10.1007/s00253-018-9275-z) contains supplementary material, which is available to authorized users.

## Introduction

Yellow fever virus (YFV) belongs to the arthropod-borne *Flavivirus* genus circulating between non-human primates in the sylvatic cycle. Repeatedly, transmission vectors like *Aedes aegypti* mosquitos introduce the virus to humans in urban regions causing thousands of deaths and very serious humanitarian consequences (WHO 2016b). The lack of specific therapies for disease treatment turns vaccination into the only preventive countermeasure. Already in 1937, a very effective live-attenuated YFV vaccine was developed and manufactured in embryonated chicken eggs (Theiler and Smith 1937). Since then, the production process remained essentially unchanged through to the present day. However, when vaccination campaigns were augmented during YFV outbreaks in Angola 2016, egg-based production levels could not meet the immediate increase in vaccine demand. As a consequence, dose-sparing practices were applied to stretch vaccine supplies, but the depletion of global emergency stockpiles could not be prevented (Monath et al. 2016). Simultaneously, spreading to China that is now infested with *A. aegypti* but was so far considered free of YFV was documented (Wilder-Smith and Leong 2017). This underpins the inherent threat to public health and the urgent need to expand global YFV vaccine stockpiles (Calisher and Woodall 2016; Vasconcelos and Monath 2016). In total, the WHO estimates the global YFV vaccine demand to 1.38 billion vaccine doses for the next decades to eliminate epidemics (WHO 2016a). However, provision of a safe and fast vaccine supply based on production processes relying exclusively on pathogen-free fertilized hen’s eggs is disputable. In addition, the development of vaccines against other emerging and re-emerging viruses, such as Zika virus (ZIKV), will require additional resources. Accordingly, alternative manufacturing platforms need to be considered. This involves the use of continuous cell lines, like the adherent Vero cell (Diamond and Coyne 2017; Monath et al. 2010). However, anchorage-dependent cell growth poses serious limitations for large-scale vaccine manufacturing and process intensification (Gallo-Ramirez et al. 2015; Genzel and Reichl 2009). In contrast, suspension-adapted cell lines (like PER.C6®, AGE1.CR®, MDCK.SUS, EB66®, CAP**®,** and BHK-21 cells) showed promising cell growth in bioreactors and productivities for a wide range of viruses (Brown and Mehtali 2010; Chu et al. 2009; Genzel et al. 2013; Jordan et al. 2009; Leon et al. 2016; Lohr et al. 2009; Nikolay et al. 2017; Pau et al. 2001).

Here, we present the use of the duck embryo-derived EB66® cells as a substrate for efficient YFV and ZIKV propagation. Hollow fiber-based perfusion processes in bioreactors equipped with an on-line capacitance sensor for perfusion rate control were used to optimize cell growth and increase virus titers. Results clearly demonstrate that this platform is well-suited for process development and intensification in vaccine manufacturing, particularly for viruses that only replicate at a low cell-specific virus yield (up to 10 infectious virions per cell).

## Materials and methods

### Cell lines and viruses

EB66® suspension cells (Valneva SE) were initially maintained in EX-CELL EBx GRO-I serum-free medium (SAFC Biosciences) supplemented with 2.5 mM l-glutamine (Sigma) and cultivated in 125-mL non-baffled shake flasks (working volume 45 mL) using an orbital shaker at 37 °C, 7.5% CO_2_ atmosphere, and 150 rpm with 50-mm shaking diameter (Multitron Pro, Infors HT). For further experiments, cells from a thawed cryo-vial were directly adapted to growth in the chemically defined HyClone CDM4Avian medium (GE Healthcare) supplemented with 2.5 mM l-glutamine and passaged at least three times before performing experiments. Porcine kidney stable (PS) cells (courtesy of M. Niedrig, Robert Koch Institute, Berlin, Germany) were used for the plaque assay and were maintained in Glasgow’s minimum essential medium (GMEM) with 10% (*v*/*v*) fetal bovine serum (Gibco), 2% (*w*/*v*) FMV Peptone (LaB-M), 2 mM l-glutamine, and 2 mM pyruvate (Sigma).

The live-attenuated yellow fever virus (YFV 17D-204, produced in specific pathogen-free eggs) was kindly provided by M. Niedrig (Robert Koch Institute, Berlin, Germany). The Zika virus has been isolated from blood specimens of a PCR-positive patient in Rio de Janeiro, Brazil, and has been expanded in C6/36 insect cells (virus material by kind permission of T. S. Moreno, Fiocruz, Brazil). Infection studies were performed at a multiplicity of infection (MOI) of 0.001 based on plaque assay and at 34 or 37 °C post-infection. The sequential adaptation of YFV and ZIKV to the EB66® cell was performed at 37 °C with cells growing in shake flasks and CDM4Avian medium. Four days post-infection, 70 μL of the cell broth was transferred to the subsequent shake flask. Further passaging was carried out with 50 μL every 3 days to select for fast-propagating viruses.

### Virus quantification

Infectious virus titers were quantified by plaque assay using PS cells with a coefficient of variation of 15% as described previously (Nikolay et al. 2017). Cell-specific virus yields (CSVY) were calculated as:

1 $$ CSVY=\frac{C_{vir,\mathit{\max}}-{C}_{vir,\mathit{\operatorname{inf}}}}{X_{tot,\mathit{\max}}} $$

with the maximum virus concentration (*C*_*vir*, *max*_), the virus concentration input for infection (*C*_*vir*, *inf*_), and the total maximum cell concentration (*X*_*tot*, *max*_) obtained up to the time point of highest virus titer.

Viral RNA (vRNA) molecules were measured by real-time reverse transcription quantitative PCR (RT–qPCR) with coefficient of variations of 4% CV for YFV and 3% CV for ZIKV. After cell centrifugation and washing with PBS, intracellular vRNA was obtained by cell lysis using sonication (15 s, 90% amplitude, 80% pulse time, 4 °C pre-cooled VialTweeter with UP200St controller, Hielscher Ulrasonics). Extracellular vRNA was obtained from virus particles in the cleared cell culture fluid using NucleoSpin RNA Virus kit (Macherey & Nagel) according to the manufacturer’s instructions. A set of primers and probes specific for YFV and ZIKV were applied as described previously (see Table S1) (Domingo et al. 2012; Lanciotti et al. 2008). To determine vRNA concentration, reference standards were generated by in vitro transcription. Therefore, seed YFV and seed ZIKV genomic RNA were reverse transcribed using RevertAidH Minus RT (Invitrogen) following manufacturer’s instruction, and subsequently amplified using Phusion DNA Polymerase with primers containing restriction sites for Xbal in the forward primer and BamHI in the reverse primer, respectively. The PCR products were purified from agarose gels using the QIAquick gel extraction kit (Qiagen) and cloned into a pUC19 plasmid using a T4-DNA ligase (NEB). After plasmid transformation into competent *E. coli* DH5α cells, positive clones were selected in a blue–white screening using IPTG and *β*-X-Gal and stored in glycerol stocks at − 80 °C. Plasmids were isolated using InnuPrep Plasmid mini Kit (Analytik Jena) and sequence integrity was confirmed by its PCR product (agarose gel migration distance and Sanger-based sequencing). A T7 promotor sequence was introduced to the forward primers of the reference RNA standards, subsequently amplified in vitro, and transcribed with a TranscriptAid T7 High Yield Transcription kit (Thermo Scientific). Target RNAs were purified using NucleoSpin RNA Clean-up (Macherey & Nagel) and the concentration was determined by spectrophotometry (NanoQuant Infinity 200Pro, Tecan). Amplicon fragment lengths of 89 bp for YFV and 77 bp for ZIKV with an average mass of 340 Da/bp enabled the correlation to vRNA copy numbers (adapted and modified from Frensing et al. 2014). The RT–qPCR was performed with a one-step QuantiNova Probe RT-PCR Kit (Qiagen) and the Rotor-Gene Q (Qiagen) following the manufacturer’s recommendation.

### Pseudo-perfusion and perfusion bioreactor cultivations

Pseudo-perfusion cultivations, as a scale-down model for high cell density cultivations, were performed in non-baffled shake flasks. The cell broth was transferred once or twice a day into a falcon tube and centrifuged at 300*g* for 5 min at room temperature. A total of 70–90% of the cleared supernatant was replaced with pre-warmed basal growth medium, before the cell pellet was re-suspended and transferred back into the shake flask.

Stirred tank bioreactor cultivations of EB66® cells were conducted in a 1-L glass bioreactor (BioStat B Plus, Sartorius) with a working volume of 700 mL. Cells were inoculated at cell concentrations of 3–7.9 × 10^5^ cells/mL and grew in either GRO-I or CDM4Avian medium. Bioreactor vessels were equipped with one pitched blade impeller (ratio diameter_vessel_/diameter_impeller_ of 1.5) operating at 100 rpm. Cells were pulsed-aerated with pure oxygen through a 20-μm micro-sparger to a pO_2_ set-point of 80% with a maximum flow rate of 0.3 L/min. Prior inoculation, pH values were set to 7.4 by CO_2_ sparging and lowered to pH 7.2 when perfusion units were operated. Either the tangential flow filtration (TFF) or the alternating tangential flow filtration (ATF2, Refine) hollow fiber-based perfusion system was connected to the bioreactor vessel. To monitor weight changes, the set-up was placed on an electronic balance. While the permeate pump rate was either adjusted manually or controlled based on the on-line conductivity signal, the feed pump rate was attuned by the BioStat control unit to maintain a pre-defined weight of the total set-up, and consequently the working volume.

#### Cryo-preservation and bioreactor inoculation

Cells for cryo-preservation were grown in the perfusion culture ATF#2 and harvested at 1.9 × 10^7^ cells/mL with about 95% viability. A total amount of 8.5 × 10^8^ cells was centrifuged at 300*g* for 5 min at 4 °C. The cell pellet was re-suspended in 7.5 mL of the supernatant and conditioned with an equal volume of pre-chilled freeze medium at 4 °C (CDM4Avian medium supplemented with 2.5 mM l-glutamine, 0.2 M sucrose, and 20% (*v*/*v*) DMSO). Cryo-bags (CryoMACS freezing bag 50, Miltenyi Biotec) were held on ice and filled with 15 mL of the cell suspension. Bags were subsequently step-wise frozen at − 20 °C for 12 h, and then stored at − 80 °C in an ultra-low deep-freezer for 10–13 days until use. For direct bioreactor inoculation, a cryo-bag was thawed in a water bath at 37 °C until a small ice pellet remained. Before connecting to the autoclaved bioreactor via sterile connectors, the bag was turned upside down several times to gently disperse the cell broth. The double-jacket glass bioreactor was chilled to 10 °C with cold process water and cells were added drop-wise into the vessel. Cold basal growth medium was then drop-wise filled into the bioreactor. Temperature control and stirring were initiated when impeller blades were half covered by medium, and process values slowly increased to set points.

#### Tangential flow filtration system

The TFF unit was connected by two dip-tubes to the bioreactor. In the external circulation loop, a centrifugal pump (Puralev® 200MU, Levitronix) operated at 1200 rpm and pushed the cell culture broth through a vertically positioned PES hollow fiber membrane (0.2 μm pore size, 1300 cm^2^, Spectrum Labs). The cultivation was performed with GRO-I medium. After 3 days, the perfusion mode was initiated using perfusion medium A (GRO-I + 4 mM l-glutamine) for cell growth and perfusion medium B (medium A + 3 g/L glucose) for virus production. Perfusion rates were adjusted manually based on off-line metabolite measurements to maintain glucose concentrations above 1 g/L.

#### Alternating tangential flow filtration system

The alternating tangential flow filtration (ATF 2, Refine) unit with a PES hollow fiber membrane (0.2 μm pore size, 470 cm^2^, Spectrum Labs) was connected via a single dip-tube to the bioreactor. The diaphragm pump operated at a flow rate of 0.5 L/min and increased to 0.8 L/min after the first day. Pump parameters, set-points, and process ranges were used as given by the supplier. The bioreactor was equipped with a capacitance probe (Incyte, Hamilton) for on-line monitoring of cell concentrations. The basal growth medium, as well as the perfusion medium was CDM4Avian medium supplemented with 2.5 mM glutamine. In the first ATF run (ATF#1), the perfusion process was started when the glutamine concentration reached 1 mM. Perfusion rates were then adjusted manually to maintain the glutamine concentration while avoiding glucose levels below 1 g/L. Therefore, viable cell concentrations (*X*_*v*_) and substrate consumption rates of the previous sampling interval (*q*_*S*_) were determined to calculate the present perfusion rate (*Q*_*pres*_) as:

2 $$ {Q}_{pres}=\frac{X_v\ {V}_w\ {q}_S}{s_0} $$

with the working volume (*V*_*w*_) and the substrate concentration of the perfusion medium (*s*_0_). To meet the expected metabolite demand until next sampling point, the last specific cell growth rate (*μ*) was determined and prospective perfusion rate (*Q*_*prosp*_) was calculated:

3 $$ {Q}_{prosp}=\frac{X_v\ {e}^{\upmu \mathrm{t}}\ {V}_w\ {q}_S}{s_0} $$

with initial parameters of *μ* = 0.035 h^−1^, *q*_*GLN*_ = 6 pM/cell/h, or *q*_*GLC*_ = 28 pM/cell/h (based on Vázquez-Ramírez et al. 2018). Perfusion rates *Q*_*pres*_ were set in the control unit of the bioreactor system with a time-dependent linear increase to *Q*_*prosp*_ and re-adjusted after each sampling time point. The media osmolality was measured in all cultivations. In the ATF#1 run, the osmolality was re-adjusted with sodium chloride (5.13 M) to 285 mmol/kg or higher when the osmotic pressure dropped below 265 mmol/kg. The second ATF run (ATF#2) was performed at a fixed cell-specific perfusion rate (CSPR), which was based on:

4 $$ CSPR=\frac{D_{perf}}{X_v} $$

and defined by perfused media volume rate (*D*_*perf*_) per viable cell. The CSPR was set to 17 pL/cell/day and perfusion rates were adjusted automatically based on on-line permittivity signals, as described in the following chapter. Manual intervention encompassed the supplementation of 112 mg l-methionine to 0.75 mM at day 4. The third ATF run (ATF#3) started 15 h post-inoculation with a CSPR of 34 pL/cell/day and without any manual interventions. Infections of the ATF runs were carried out at targeted cell concentrations exceeding 5 × 10^7^ cells/mL and at first indications of a reduction in the cell growth rate.

#### On-line permittivity signal for cell-specific perfusion rates

**Fig. 1:**
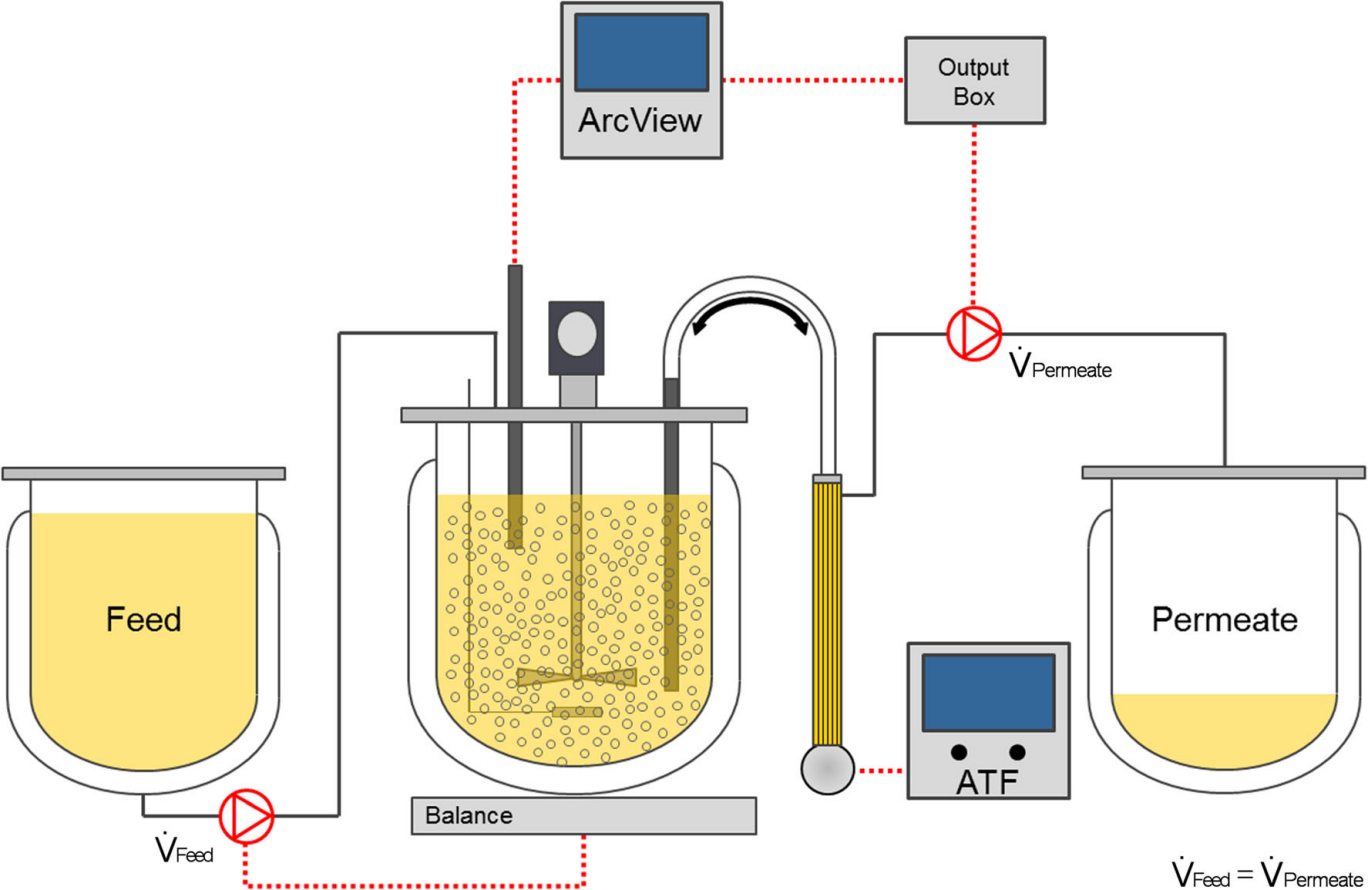
Scheme of the perfusion bioreactor set-up using a capacitance probe for on-line monitoring of cell growth. In the ArcView controller, the permittivity signal is converted to an analog signal (4–20 mA) and used to control a peristaltic permeate pump to maintain defined cell-specific perfusion rates

The on-line multi-frequency capacitance probe operated at a frequency range of 1–10 MHz and was connected to a controller (ArcView Controller 265, Hamilton) with a data acquisitioning time of 6 min. The measured capacitance was converted automatically into a permittivity signal. While cells were growing, both the total viable cell volume (VCV) and the viable cell concentration (VCC) showed first-order correlations with the permittivity signal (correlation coefficient > 0.99; data not shown). For perfusion rate control, the slope, describing a specific cell factor, was used to calibrate the ArcView controller. Cell factors of 1.1, 1.7, and 1.8 were determined for the three ATF runs. The on-line permittivity signal was then configured in the advanced software setting of the ArcView controller to an output signal, which was forwarded to an analog 4–20 mA output box (Hamilton). This signal was used to control a peristaltic permeate pump (120 U, Watson-Marlow) for media perfusion (Fig. 1).

### Sampling and analytics

EB66® cells grew as single cells and loose cell clumps in shake flask cultivations. Thus, samples were first dispersed by manual pipetting prior cell counting (ViCell XR, Beckman Coulter). Cell samples from stirred tank bioreactor cultivations, whereas formed stronger aggregates, which were treated enzymatically. In brief, 200 μL of the cell broth was added to 200 μL porcine trypsin (5000 U/mL, Gibco) and incubated at 600 rpm for 10 min and 37 °C (Thermomixer, Eppendorf). The reaction was stopped with 200-μL fetal calf serum before cell counting in analytical triplicates. Mean cell diameters were determined from ViCell size distribution measurements with a total number of 100 images per single measurement. Metabolite concentrations were measured with a Bioprofile 100 Plus (Nova Biomedical) and osmotic pressure was measured with the Vapro 5520 (Wescor Vapro). Amino acid analysis was carried out on an Acquity H-Class UPLC instrument (Waters).

## Results

### YFV and ZIKV infection studies in EB66® cells

**Fig. 2:**
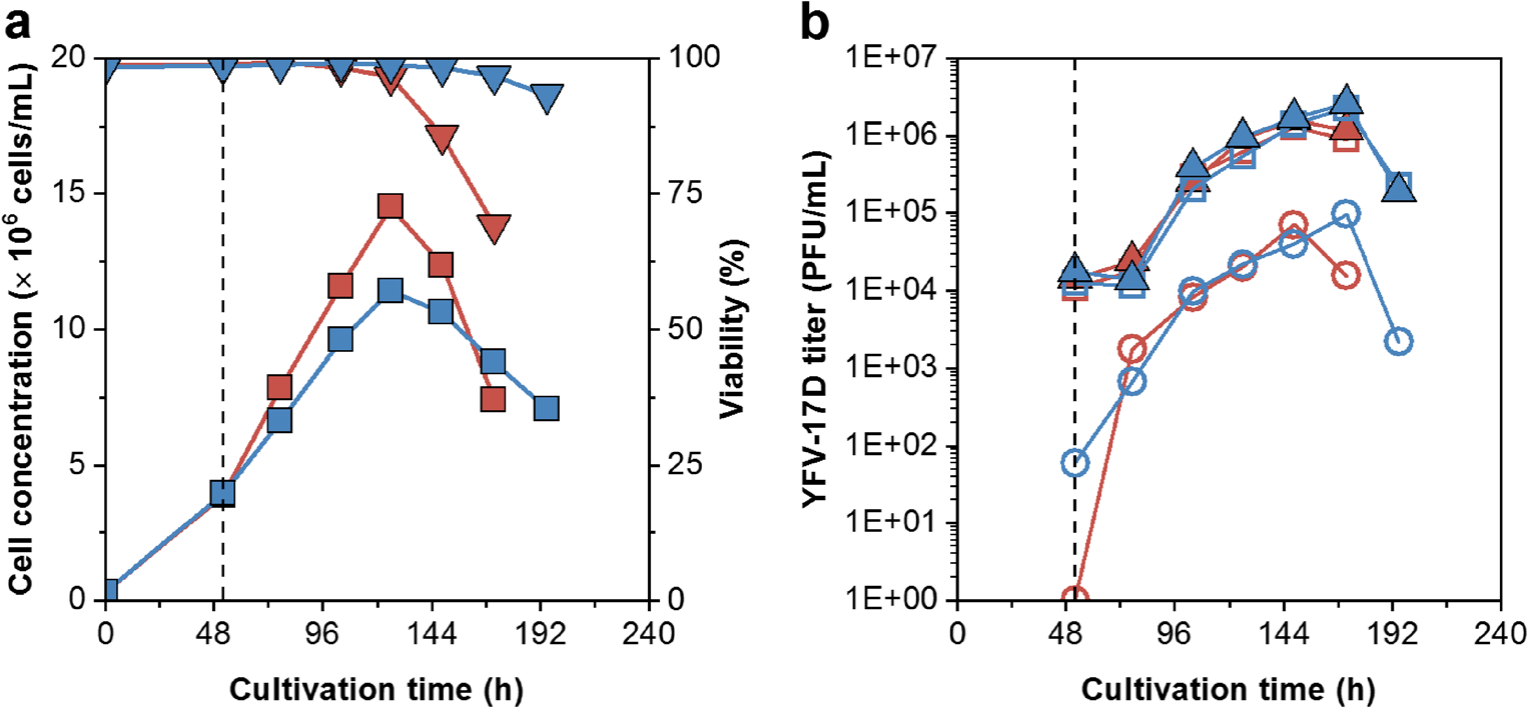
Shake flask infection studies of EB66® cells in CDM4Avian medium demonstrating the impact of a temperature shift from 37 °C (red) to 34 °C (blue) at time of yellow fever virus (YFV) infection (dotted vertical line) in GRO-I medium on **a** cell concentration (■),viability (▼) and on **b** intracellular (○) and extracellular infectious virus titer (□) in comparison to the total infectious virus titer (▲)

**Fig. 3:**
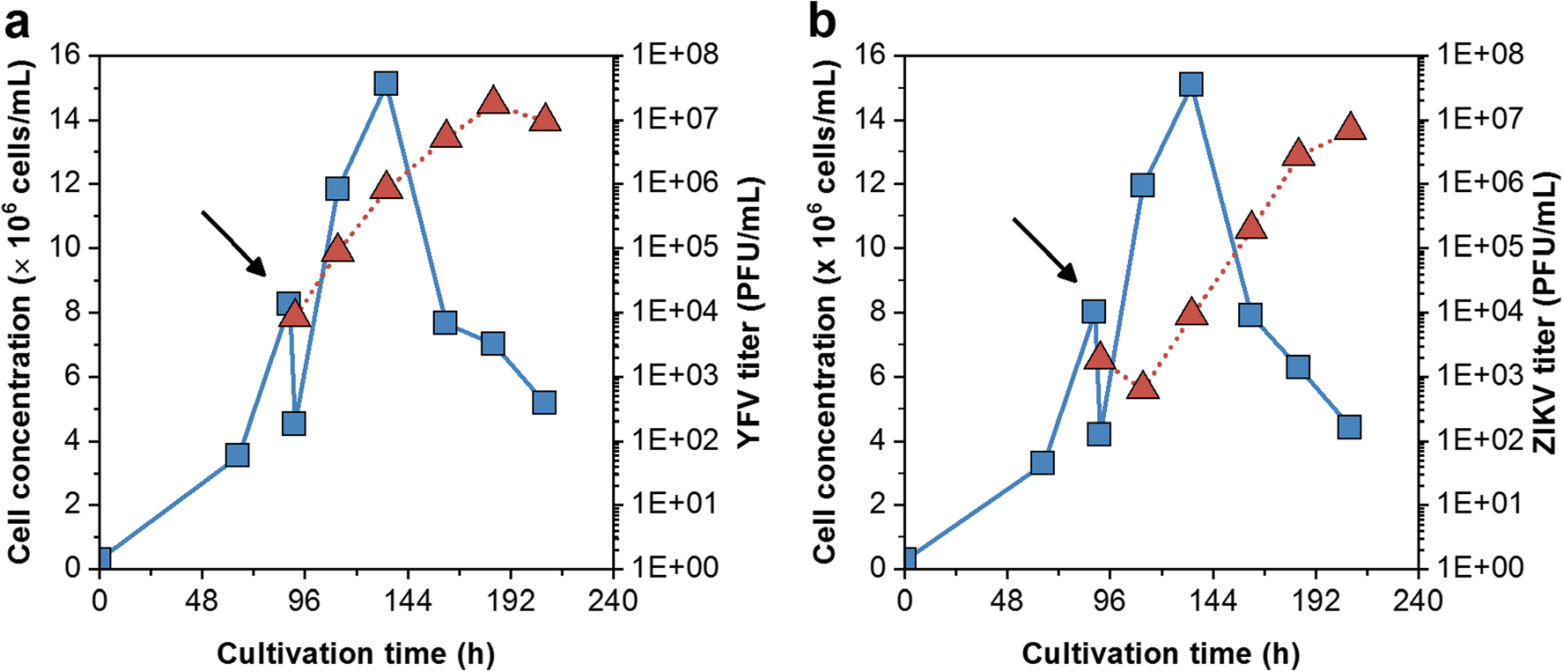
Shake flask infection studies of EB66® cells in CDM4Avian medium with **a** yellow fever virus (YFV) and **b** ZIKA virus (ZIKV). Prior infection, the cell culture was split into two shake flasks, filled-up with fresh medium and infected. Cell concentration (), virus titers (). Arrows indicate time of infection

**Fig. 4:**
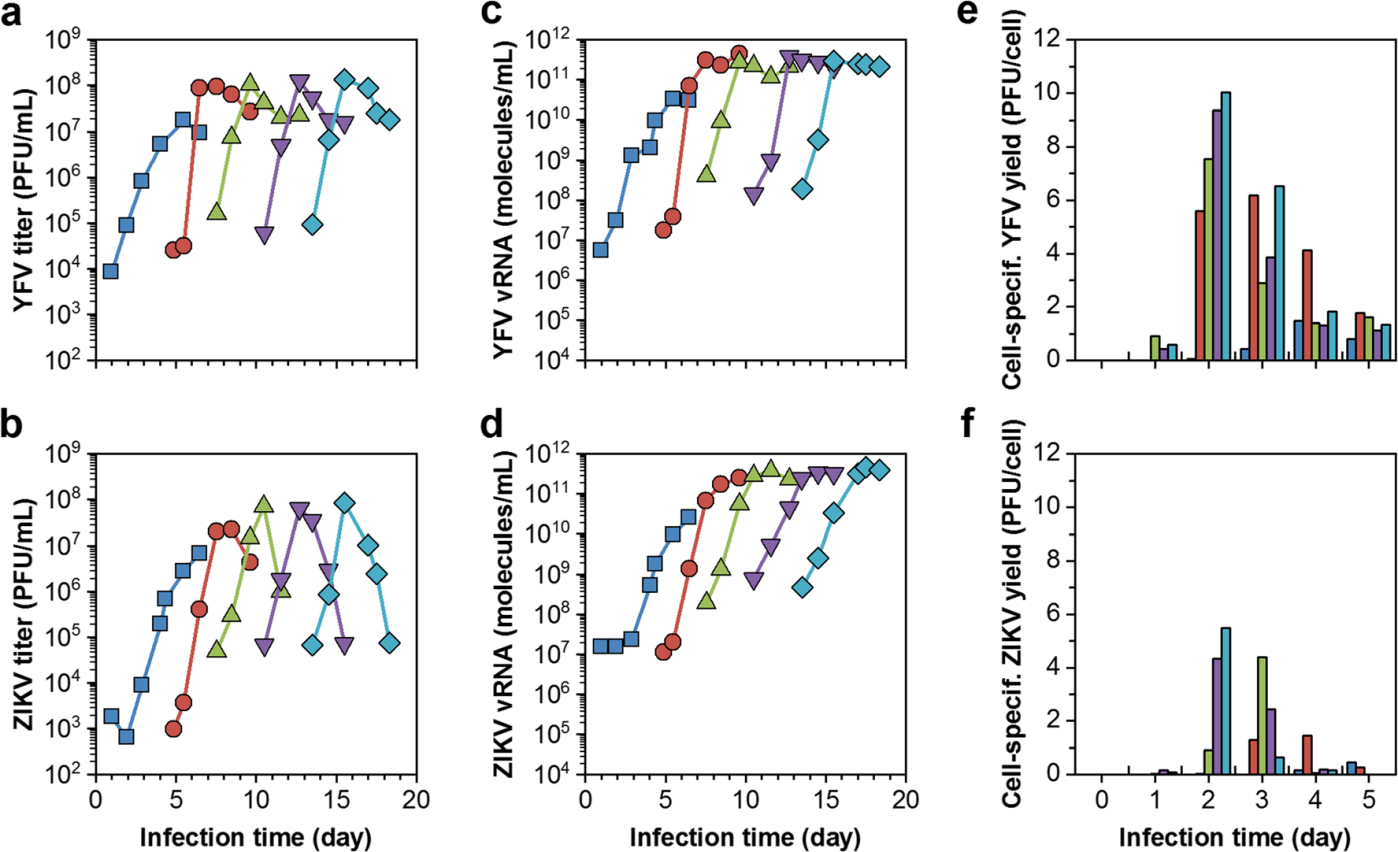
Sequential passaging of yellow fever virus (YFV) and ZIKA virus (ZIKV) in EB66® cells growing in CDM4Avian medium and shake flasks. Each passage is indicated by a different color (blue = 1st passage, red = 2nd passage, green = 3rd passage, purple = 4th passage, turquois = 5th passage). **a**, **b** Infectious virus titers, **c**, **d** vRNA concentrations, and **e**, **f** cell-specific virus yields for YFV and ZIKV, respectively

EB66® cells grew in shake flasks and GRO-I medium to average cell concentrations of 1.4 × 10^7^ cells/mL with a population doubling time (t_D_) of 19 h. Infection studies with YFV resulted in maximum titers of 1.3 × 10^6^ PFU/mL. Attempts for virus yield improvements included a temperature shift at time point of infection from 37 to 34 °C. This did not increase titers, whereas cell disruption released cell-associated and intracellular infectious virus material. By that, YFV titers increased slightly by 15% at 34 °C and 22% at 37 °C (Fig. 2). With the cell adaptation to the newly developed chemically defined CDM4Avian medium, cell concentrations increased routinely to about 2 × 10^7^ cells/mL with a t_D_ of 17 h (not shown here). Infection studies with YFV and ZIKV resulted in 1.8 × 10^7^ and 6.8 × 10^6^ PFU/mL, respectively, leading to cell-specific virus yields of 1.2 and 0.5 PFU/cell (Fig. 3). Moreover, YFV and ZIKV seed strains were subcultured over five passages in EB66® cells. Over the time course of virus adaptation, YFV and ZIKV titers increased to maximum titers of 1.4 × 10^8^ and 8.5 × 10^7^ PFU/mL, respectively. The time to reach final titers decreased from 4 to 2 days for YFV and from more than 6 to 2 days for ZIKV (Fig. 4a, b). RT–qPCR was used to quantify vRNA concentrations in the supernatant. For YFV, vRNA levels correlated with PFU titers and peaked at 2.9 × 10^11^ molecules/mL. In contrast, ZIKV levels increased beyond the time point of the maximum infectious titer to 4.6 × 10^11^ molecules/mL (Fig. 4c, d). In total, by each passage throughout virus adaptation, cell-specific virus yields increased to finally 10 PFU/cell (YFV) and 5.5 PFU/cell (ZIKV), respectively. With each passage, the highest ratio was obtained faster and correlated with maximum virus titers (Fig. 4e, f). Furthermore, for passage five (last passage) and maximum virus titers, the ratio of RNA concentration to PFU was 2.1 × 10^3^ vRNA/PFU and 0.4 × 10^3^ vRNA/PFU for ZIKV, respectively.

### Perfusion cultivations in serum-free and chemically defined medium

To maximize cell concentrations improving virus yields, small-scale pseudo-perfusion cultivations were conducted in GRO-I and CDM4Avian medium. Batch-wise media exchanges of the centrifuged, cell-free supernatant were performed daily and led to increased cell concentrations of 2.5 × 10^7^ cells/mL for both media, whereas cell-specific growth rates increased strongly over 8 days (Fig. S1). Glucose and glutamine levels were maintained at levels higher than 1 g/L and 1 mM, respectively. A strong formation of a stirring ring was observed at the plastic wall. It contained of approximately 10% of the total amount of cultivated cells (9 × 10^7^ cells) at a viability of about 60%.

To transfer the small-scale cultivation into membrane-based perfusion bioreactor systems, EB66® cells were cultivated in GRO-I medium and a bioreactor coupled to a TFF cell retention unit. Cells were infected with a non-adapted YFV seed at day 6, and cells grew continuously to 5.7 × 10^7^ cells/mL over 9 days. Thereby, an initial t_D_ of 18 h was observed during the first 3 days in the batch phase and increased to a t_D_ of 35 h when the process mode was changed to perfusion. Maximum virus titers of 3.1 × 10^6^ PFU/mL were achieved 4 days post-infection (Fig. S2). Follow-up experiments using CDM4Avian medium, however, resulted in a decrease of cell concentrations and viabilities below 80% when the centrifugal pump was started (data not shown).

**Fig. 5:**
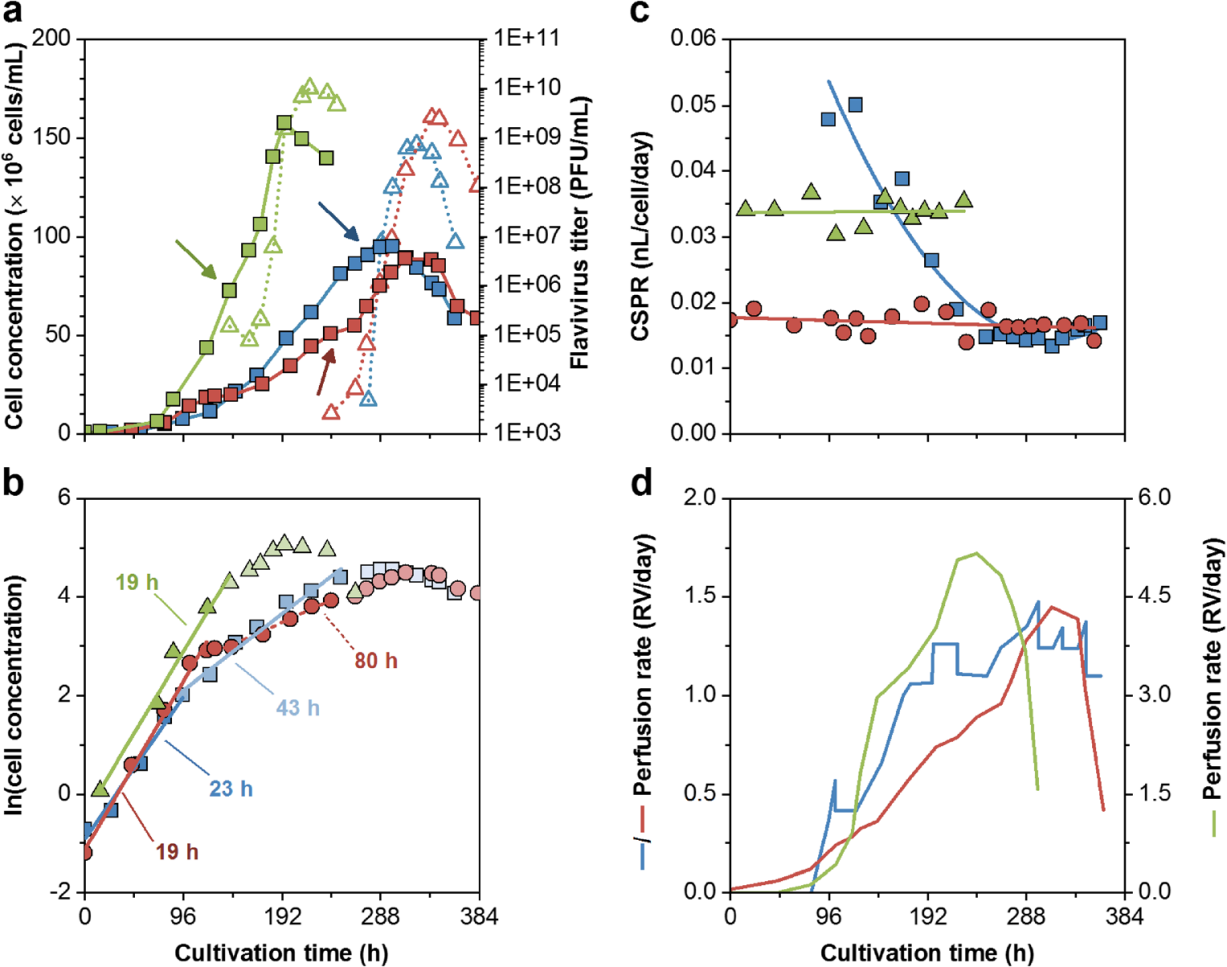
Comparison of three high cell density cultivations using ATF units. Perfusion rates were either adjusted manually based on off-line measured cell and metabolite concentrations (ATF#1 = blue), or controlled based on measurements of the on-line capacitance probe using fixed cell-specific perfusion rates (ATF#2 = red; ATF#3 = green). **a** Cell concentration (■) and virus titers (△; yellow fever virus (YFV = blue), ZIKA virus (ZIKV = red, green)). **b** Logarithmic growth profiles and linear fitting of the batch phase (blue) and the perfusion phases of ATF#1 (light blue), ATF#2 (red), and ATF#3 (green). **c** Cell-specific perfusion rates (CSPR) with trend lines. **d** Reactor volume exchange rates per day (for ATF#3 secondary y-axis). Arrow indicates the time point of infection

**Fig. 6:**
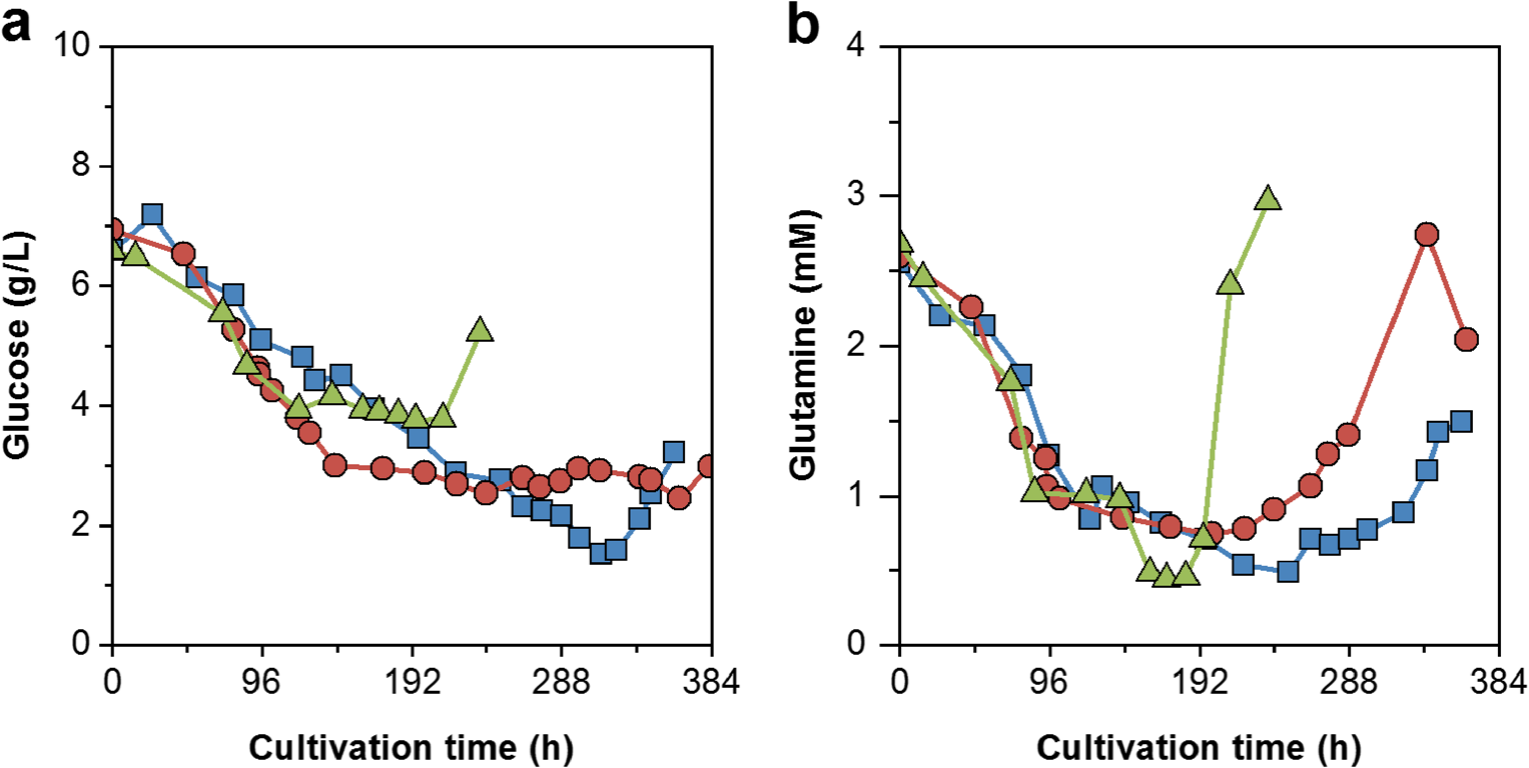
Metabolite profiles for high cell density cultivations using ATF units. **a** Glucose and **b** glutamine concentrations for ATF#1 (), ATF#2 (), and ATF#3 () perfusion runs

**Fig. 7:**
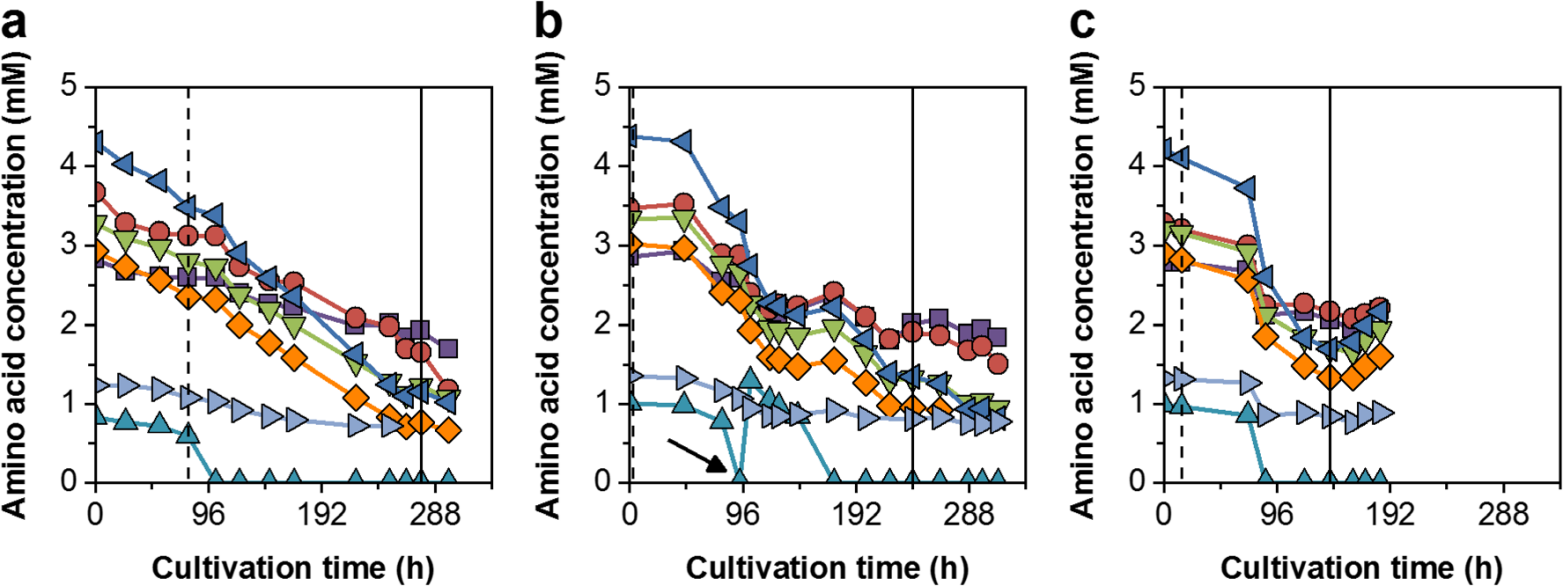
Essential amino acid profiles for high cell density cultivations using ATF units of **a** ATF#1, **b** ATF#2, and **c** ATF#3 until on-set of cell death. Leucine (), lysine (), valine (), isoleucine (), threonine (), phenylalanine (), methionine (). Dotted line indicates start of perfusion phase; solid line indicates time point of infection; arrow indicates methionine supplementation

To test an alternative hollow-fiber membrane-based perfusion system, an ATF unit was coupled to the bioreactor systems. The subsequent ATF perfusion cultivation (ATF#1) facilitated cell growth in CDM4Avian medium. Thereby, cells grew continuously with a t_D_ of 23 h for 3 days in batch mode. When the perfusion process was started at day 4, cell growth slowed down to a t_D_ of 43 h. After 12 days, cell concentrations increased to 9.1 × 10^7^ cells/mL, and cell was subsequently infected with the EB66®-adapted YFV virus (5th passage). Final virus titers peaked within 2 days with 7.3 × 10^8^ PFU/mL corresponding to a cell-specific virus yield of 8 PFU/cell (Fig. 5a, b). As perfusion rates were adjusted manually to keep glucose concentrations above 1 g/L and glutamine levels at 1 mM (Fig. 6), fluctuations of the daily reactor volume exchange rate (RV/d) were observed. Initially, 0.5 RV/d were set and later increased step-wise to 1.5 RV/d. Thereby, the CSPR decreased constantly from 50 to 15 pL/cell/day (Fig. 5c, d). Based on the total media consumption of 7.7 L, a media volume-specific cell yield of 1.2 × 10^7^ cells/mL_Medium_ was achieved. The total volumetric YFV productivity was 4.5 × 10^9^ PFU/L/day (Table 1). Later, amino acid analysis revealed a simultaneous depletion of methionine when cell growth slowed down (Fig. 7a).

**Table 1:** Process data and flavivirus production in EB66® cells cultivated in different perfusion runs

Perfusion system	Medium	Medium strategy	Max. cell concentration	Spent medium^a,b^	Cell-specific perfusion rate	Volume-specific cell yield^b^	Virus strain	Virus titer	Cell-specific virus yield	Volumetric virus productivity^b^
			cells/mL	L	pL/cell/day	cells/mL_Medium_		PFU/mL	PFU/cell	PFU/L/day
TFF#1	GRO-I	Manual > 1 g/L Glc	5.7 × 10^7^	6.1	125–12	7.6 × 10^6^	YFV^c^	3.1 × 10^7^	0.05	3.2 × 10^7^
ATF#1	CDM4Avian	Manual 1 mM Gln and > 1 g/L Glc	9.5 × 10^7^	7.7	50–15	1.2 × 10^6^	YFV	7.3 × 10^8^	8	4.5 × 10^9^
ATF#2	CDM4Avian	Fixed CSPR	8.9 × 10^7^	6.6	17	1.3 × 10^6^	ZIKV	2.6 × 10^9^	30	1.8 × 10^10^
ATF#3	CDM4Avian	Fixed CSPR	1.6 × 10^8^	12.6	34	1.3 × 10^6^	ZIKV	1.0 × 10^10^	65	6.0 × 10^10^

^a^Until maximum virus titer was reached

^b^Includes basal batch growth medium and perfusion medium

^c^YFV harvested from Vero cells

**Fig. 8:**
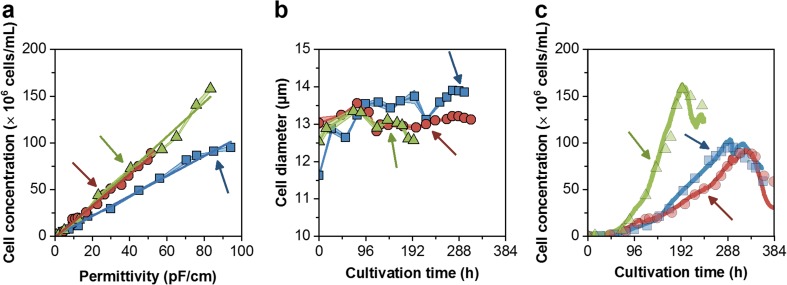
**a** Correlation of off-line measured viable cell concentrations with on-line permittivity signal during cell growth (including infection phase, indicated by arrows) in ATF#1 (), ATF#2 (), and ATF#3 () runs. The slope of the respective linear regressions corresponds to 1.1 (ATF#1), 1.7 (ATF#2), and 1.8 (ATF#3). **b** Changes in cell diameter during growth. **c** On-line monitoring of cell concentrations (solid line) compared to off-line ViCell measurements (symbols) for all perfusion cultivations. Average of three analytical replicates with standard deviation as shaded background

For the second (ATF#2) and third ATF run (ATF#3), the on-line capacitance probe was used to maintain constant CSPRs. The capacitance signal showed a linear correlation of the permittivity signal to the viable cell concentration determined by ViCell off-line measurements. Thus, cell growth could be monitored accurately at high cell densities throughout the cultivation (Fig. 8c). This allowed the control of perfusion rates at defined CSPRs. The perfusion process started from time point of inoculation and was automatically controlled. For the first trial (ATF#2), a CSPR of 17 pL/cell/day was set and cells grew at t_D_ of 19 h. To avoid methionine depletion (ATF#1), the medium was supplemented with this essential amino acid after 4 days. However, amino acid analysis revealed the depletion of methionine shortly before supplementation (Fig. 7b); the decrease in cell-specific growth rate could not be overcome and t_D_ increased to 80 h. When cells reached 5.1 × 10^7^ cells/mL, they were infected with EB66®-adapted ZIKV (5th passage) and continued growing to maximum concentrations of 8.9 × 10^7^ cells/mL. ZIKV titers increased over 4 days to 2.6 × 10^9^ PFU/mL leading to a cell-specific virus yield of 30 PFU/cell. With a total use of 6.6 L medium, this corresponded to a volumetric virus productivity of 1.8 × 10^10^ PFU/L/day (Fig. 3a, Table 1).

For the second cultivation with a CSPR of 34 pL/cell/day, the perfusion process (ATF#3) was started directly from a cryo-bag. Prior to this, thawing procedures of cryo-bags at different cell stock concentrations (2 × 10^7^, 4 × 10^7^, and 6 × 10^7^ cells/mL) were tested by inoculating shake flasks. Experimental data obtained for recovery and cell growth (data not shown) suggested a direct inoculation into an empty, pre-chilled bioreactor vessel before cold growth medium was added. Thereby, DMSO concentrations were diluted to about 0.2% and the cultivation started with 7.9 × 10^5^ cells/mL. In total, 65% of the cryo-preserved cells were recovered with a total viability of 94%. After a lag phase of 1 day, cells recovered and achieved a similar maximum specific growth rate. However, cell growth decreased from the initial t_D_ of 19 h to about 40 h after 5 days of cultivation while methionine (not additionally supplemented) was depleted after about 4 days (Fig. 7c). Nevertheless, infection of cells at 7.3 × 10^7^ cells/mL with EB66®-adapted ZIKV (5th passage) resulted in increasing cell concentrations to a maximum of 1.6 × 10^8^ cells/mL 2 days post-infection (8 days total process time) with highest ZIKV titers of 1.0 × 10^10^ PFU/mL 3 days post-infection (Fig. 5). This corresponded to a cell-specific virus yield of 65 PFU/cell. With a total consumption of 12.6 L medium, a volumetric virus productivity of 6.0 × 10^10^ PFU/L/day (Table 1) was achieved. In both automated ATF perfusion cultivations, glucose and glutamine concentrations were maintained at levels comparable to the manually adjusted ATF#1 run. The increasing concentrations of both substrates towards the end of each cultivation correlated with cell lysis and cell death indicating reduced substrate uptake rates and the release of intracellular metabolites (Fig. 6).

Finally, despite the low diameter of both viruses (about 50 nm), only a negligible amount of infectious virus particles (< 1%) was transferred to the permeate of the hollow fiber membrane (cut-off 0.2 μm).

## Discussion

### Improving cell-specific YFV and ZIKV yields in EB66® cells

Initial infection studies demonstrated EB66® cells to be permissive for YFV and ZIKV. To improve YFV titers, the process temperature was shifted at time of infection to 34 °C. This reduced cell-specific growth rate by about 20%. While cell viabilities above 93% were maintained over an extended infection period, virus titers increased only slightly. Nevertheless, lower temperatures can reduce virus inactivation (fast decrease in infectious virions) as flaviviruses are known to be relatively thermolabile. This has been confirmed by virus spiking experiments of cell-free medium, where half-life increased from 11 h (YFV) and 8 h (ZIKV) at 37 °C to 20 h and 14 h at 33 °C (unpublished data). Furthermore, at later time points of the infection phase, the release of cell-derived proteases may contribute to viral degradation. Accordingly, a temperature shift at time of infection should be considered for the optimization of live-attenuated vaccine production processes. Based on previous studies indicating poor ZIKV release from suspension cells (Nikolay et al. 2017), another approach to increase virus titers aimed at the disruption of infected EB66® cells. Surprisingly, mechanical sonication did not affect infectious virus titers in spiking experiments, but burst infected cells effectively and increased viral titers by about 22%. Thus, sonication seems to be an alternative to time consuming and degradative, repeated freeze/thaw cycles often performed in vaccine manufacturing. However, the strong accumulation of cell debris together with increasing concentrations of cellular DNA and host cell proteins may impair subsequent virus purification steps, which can cancel out the increase in yields.

For the YFV and ZIKV seeds adapted in EB66® cells, both infectious titers and vRNA copy numbers increased significantly by about one log unit. Thereby, maximum titers were already obtained 2 days post-infection. Interestingly, the ratio of vRNA to PFU at time point of maximum virus titers, corresponding roughly to the fraction of total virus particles to infectious virions, was lower for the adapted virus seeds (YFV: 2.1 × 10^3^ vRNA/PFU, ZIKV: 0.4 × 10^3^ vRNA/PFU) compared to the native material (YFV: 3.2 × 10^3^ vRNA/PFU, ZIKV: 3.8 × 10^3^ vRNA/PFU). Notably, the ratio of vRNA to PFU dropped for ZIKV by almost one order of magnitude after adaptation to EB66® cells. The fact that sequential virus passaging did not only increase the total number of virions (vRNAs) produced by the cells but also total infectivity is most likely due to more efficient intracellular virus replication, packaging, and release. In addition, an improved thermostability of infectious virions may also play a role. Virus sequencing can help to understand these mechanisms for virus adaptation on a genomic level.

Overall, with an increase in cell-specific virus yields to 10 PFU/cell for YFV and 5.5 PFU/cell for ZIKV, respectively, the EB66® cell line is a viable option for flavivirus production compared to other continuous cell lines, i.e., adherent Vero cells or suspension-adapted BHK-21 cells. For the latter, depending on isolates and virus–cell adaptation, ZIKV yields in the range of 1–48 PFU/cell were obtained (Nikolay et al. 2017).

### Perfusion processes and the use of a capacitance probe for perfusion rate control

Suspension growth of EB66® cells is highly advantageous in comparison to adherent cell growth and facilitates process intensification by high cell density cultivation. While initial scale-down pseudo-perfusion studies with EB66® failed due to a strong coagulation of cells at the shake flask wall, the obtained cell concentration of 2.5 × 10^7^ cells/mL served as a reference value for following membrane-based perfusion cultivations as a proof-of-concept study for process intensification.

In a first approach, cells were cultivated in GRO-I medium using a TFF perfusion bioreactor. Cells grew continuously after YFV infection for another 3 days to 5.7 × 10^7^ cells/mL. Although the infection with a non-adapted YFV seed resulted only in relatively low virus titers of 3.1 × 10^6^ PFU/mL, the TFF perfusion set-up clearly demonstrated its potential to achieve even higher cell densities at bioreactor scale. The follow-up experiment using CDM4Avian medium led to decreased cell concentrations and viabilities once the centrifugal pump has been started. Microscopic observations revealed the dispersion of cell clumps into single cells when the centrifugal pump was started. Various approaches to cultivate cells at the lowest pump speed (800 rpm) or even without a hollow-fiber membrane in the re-circulation loop could not overcome this issue. Although the used Puralev® pump is designed for low shear-stress recirculation, it did not support cell growth in combination with the chemically defined CDM4Avian medium.

In contrast, ATF perfusion bioreactors using a low shear diaphragm pump enabled cell growth in CDM4Avian medium. Here, the smallest ATF2 system was used which is, in principle, scalable up to 1000-L cultivations with the ATF10 module. The perfusion rate was first adjusted manually to maintain minimum glucose and glutamine concentrations (Clincke et al. 2013; Genzel et al. 2014; Vázquez-Ramírez et al. 2018). However, this needed permanent re-adjustments due to varying cell growth and metabolite uptake rates. Nevertheless, despite significant fluctuations in perfusion rates, concentrations up to 9.5 × 10^7^ cells/mL were achieved for infected EB66® cells in the ATF#1 run. In combination with EB66®-adapted YFV seeds, this resulted in increased virus titers and a cell-specific virus yield of 8 PFU/cell (comparable to high-yield shake flask batches).

Based on these results, two ATF runs for ZIKV production were carried out with an automated perfusion rate control to avoid manual adjustments and operate under more defined conditions. A similar concept was described by Dowd et al. (2003), who demonstrated the feasibility of such a process control loop for CHO cell cultivations reaching final concentrations of about 5 × 10^6^ cells/mL. The approach was later adapted to adjust cell concentrations by controlled cell bleeding using a capacitance probe (Konstantinov et al. 2006). For all three ATF runs performed in present study, the on-line permittivity signal was first correlated to the total VCV, as the capacitance probe output is proportional to the polarized volume fraction enclosed by an intact membrane (Harris et al. 1987). However, despite some changes in cell diameters over the time course of cultivations, the coefficient of determination (*R*^2^) for estimation of the VCC was also high (> 0.99%). And, further analysis based on the sum of squared residuals calculated for both linear regressions that were normalized to the permittivity signal for VCV and VCC, revealed no significant differences between both estimations (F-test, *P* ≤ 0.05). Taking into account that cell concentration measurements are typically performed in routine production, the on-line control was set to measure VCC. As slopes of the regression line slightly vary between runs, first off-line measured cell concentrations could be taken for immediate re-adjustments of VCC estimations from the permittivity signal. However, causes for the batch-to-batch variation of this signal (variations in slope at 1.1–1.8) are currently not fully understood and need further investigation.

For both perfusion cultivations, the higher CSPR led to never reported EB66®cell concentrations of 1.6 × 10^8^ cells/mL. In combination with the stable CSPR profile, this clearly underlined the successful use of a capacitance probe for perfusion rate control.

In general, CSPRs can vary strongly between bioprocesses and are typically chosen in the range of 50–500 pL/cell/day depending on the feeding profile (Konstantinov et al. 2006). Previous perfusion cultivations with the avian AGE1.CR.pIX® cell line were conducted in manual mode at about 60 pL/cell/day, and concentrations of 5.0 × 10^7^ cells/mL were achieved before infection (Vázquez-Ramírez et al. 2018). Here, the use of the CDM4Avian medium developed specifically for growth of EB66® cells and a CSPR as low as 17 pL/cell/day facilitated already high cell density conditions. By varying CSPRs, potential metabolite limitations and growth inhibition due to accumulation of lactate and ammonia (data not shown) were avoided. Cell-specific substrate uptake rates for all ATF runs showed a similar profile indicating a stable cell metabolism independent of the feeding rate. This led to comparable trends in the uptake of glucose, glutamine, and other amino acids. Assessing the total use of media for the perfusion experiments, similar ratios of cell number to medium volume were obtained for all ATF runs. Thereby, volume-specific cell yields of 1.3 × 10^6^ cells per mL of medium were obtained in ATF runs, whereas yields of shake flask batch cultivations were only slightly higher with 1.5 × 10^6^ cells per mL of medium. The reduction of medium costs is pivotal for perfusion process optimization and to compete against batch and fed-batch production processes (Konstantinov et al. 2006). Most likely, the optimal CSPR for EB66® cells as well as the maximum performance of the ATF processes established (cell concentration, virus yield, and productivity) was not reached, yet. The step-wise improvement in the design of ATF runs resulted in a more complex set-up with conclusive data, i.e., regarding cell growth performance and virus yields. However, latest for manufacturing scale operation, cell concentrations, infection conditions, cell-specific virus yield, level of impurities, and total costs should be re-evaluated again, and—based on a design of experiments approach—the final set-up identified.

Furthermore, never reported ZIKV titers of 1.0 × 10^10^ PFU/mL and increased cell-specific virus yields (65 PFU/cell) clearly indicate that ATF perfusion processes not only enable high cell concentrations but improve ZIKV replication: in contrast to shake flask cultivations, this was most likely due to controlled process conditions and the extended infection phase. For the ATF runs, cell-specific yields could be increased when infections were initiated at higher cell-specific growth rates. In addition, the shortened infection period reduced the impact of unspecific virus inactivation. Presented results indicate the absence of high cell density effects, potentially overcome by constant supply of substrates and the removal of inhibiting compounds. In comparison, cell-specific ZIKV yields exceeded those previously achieved with suspension-adapted BHK-21 cells in batch-mode (41 PFU/cell) for low cell density cultivations (Nikolay et al. 2017).

Neither YFV nor ZIKV could be harvested via the permeate outlet of the ultrafiltration modules despite the rather large pore size of the hollow fiber membranes (0.2 μm). A similar observation was not only reported in previous ZIKV studies using Repligen PES membranes at a similar cut-off (Nikolay et al. 2017), but also for influenza A virus particles using even larger pore sizes (Genzel et al. 2014). Observations that virus titers in the permeate of ATF modules decreased below 1% within 1 day post-infection (data not shown) clearly hint to virus sieving by membrane fouling (e.g., non-specific accumulation of host cell DNA, proteins, and other compounds). However, systematic studies regarding the selection of membrane cut-off and materials for virus production processes involving high cell concentrations and lysis of infected cells are still lacking. Consequently, for YFV and ZIKV, the virions should be harvested directly from the bioreactor. With respect to the thermolability of flaviviruses and the possible release of virus-degrading proteins (e.g., proteases), a continuous harvest strategy using retention devices that do not involve membrane filters such as acoustic settlers may still be an option (Petiot et al. 2011). However, scalability and retention efficiency at the very high cell concentrations achieved in this study remain to be addressed in large-scale vaccine manufacturing.

For the monitoring of virus release and the selection of the optimal virus harvest time, information obtained from the permittivity signal of the capacitance probe used for cell concentration measurements may be useful. This concerns, in particular, the characteristic frequency (f_c_), the height of the low frequency plateau (∆ε_max_), and the slope of the β-dispersion (α) recorded in all perfusion runs. However, a correlation of flavivirus production and release with f_c_, the estimated cell membrane capacitance (c_m_) or the intracellular conductivity (σ_i_), as previously described for several other viruses (Petiot et al. 2017), was not found.

To further intensify production processes, a cryo-bag was used for direct cell inoculation in the ATF#3 run. The aim was not per se a high cell density inoculation but to save time during seed train expansion and increase the flexibility to perform bioreactor cultivations. Gentle handling of the cryo-bag and careful thawing (including vessel cooling) resulted in high cell viabilities, and additional DMSO washing steps were dispensable. As another advantage, the use of well-characterized cryo-bags for inoculation may contribute to the reduction of batch-to-batch variations, ensure a more robust process performance, and help to improve product quality and quantity (Seth et al. 2013).

Overall, the combination of high cell density cultivation and EB66® cell-adapted seed virus infection can be considered a break-through in cell culture-based YFV and ZIKV production. In addition, the methods established have a great potential for the production of other viruses. The YFV harvest of the ATF#2 bioreactor cultivation (working volume 700 mL) yielded raw material equivalent to almost 10^7^ live-attenuated vaccine doses (4.74 log10 PFU/dose based on the FDA-approved YF-VAX®, WHO (2016b)) within less than 2-week operation time. Viral titers of the wild-type ZIKV isolate from ATF#3 were even higher, which make these processes extremely attractive, also for the development of inactivated ZIKV vaccines. Vero-derived ZIKV for such vaccines are currently in clinical trials for humans (ClinicalTrials.gov, numbers NCT02952833, NCT02937233, NCT02963909). Alternatives may be developed towards chimeric vaccines using a YFV backbone (Li et al. 2018) or live-attenuated ZIKV isolates (Shan et al. 2017), potentially also replicating well in EB66® cell-based perfusion cultures.

In summary, our results clearly demonstrate that EB66® perfusion cultures can be effectively used for vaccine manufacturing, in particular for flaviviruses with low cell-specific yields. Thereby, perfusion rates were controlled automatically by the successful implementation of an on-line capacitance probe, which will help to standardize future perfusion processes.

## Electronic supplementary material

ESM 1 (PDF 383 kb)
