# Supplementary material for: Process intensification of EB66® cell cultivations leads to high-yield yellow fever and Zika virus production
Source: Appl Microbiol Biotechnol. 2018 Aug 8;102(20):8725–37. doi: 10.1007/s00253-018-9275-z (PMC6153634; doi:10.1007/s00253-018-9275-z)
Supplement: Supplementary file 1 — (PDF 383 kb) [file 253_2018_9275_MOESM1_ESM.pdf]

Applied Microbiology and Biotechnology

Process intensification of EB66® cell cultivations leads to high-yield yellow fever and Zika virus production

Alexander Nikolay[1], Arnaud Léon[2], Klaus Schwamborn[2], Yvonne Genzel[1,*], Udo Reichl[1,3]

[1] Max Planck Institute for Dynamics of Complex Technical Systems, Bioprocess Engineering, Magdeburg; Sandtorstr. 1, 39106 Magdeburg, Germany
[2] Valneva SE, 6 rue Alain Bombard, 44800 Saint-Herblain, France
[3] Chair for Bioprocess Engineering, Otto von Guericke University Magdeburg; Universitätsplatz 2, 39106 Magdeburg, Germany

* genzel@mpi-magdeburg.mpg.de, Tel.: +49 391 6110-257, Fax: +49 391 6110-565

Supplementary materials

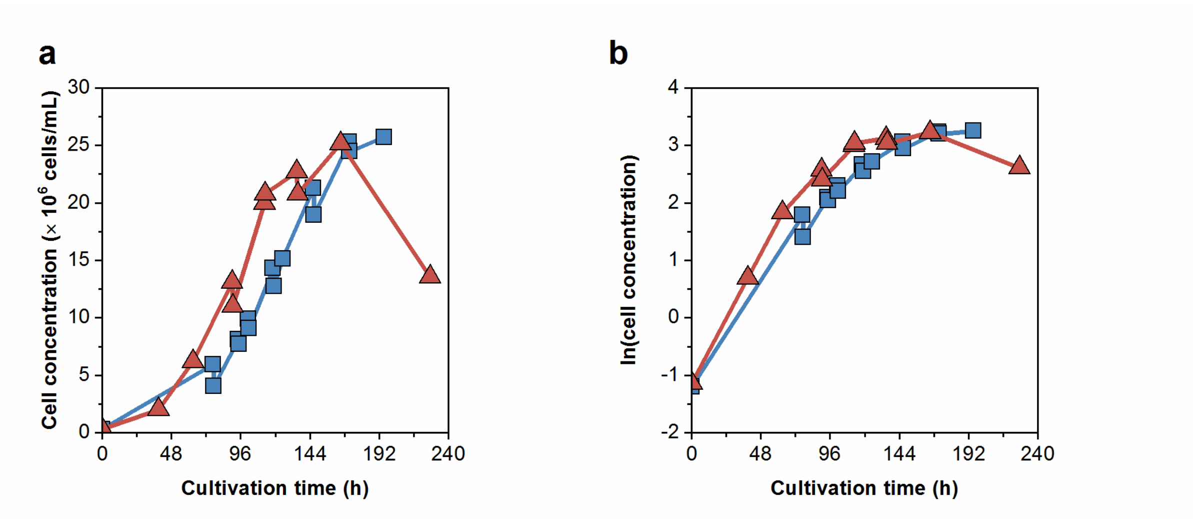

Fig. S1 (**a**) EB66® cell concentrations of a pseudo-perfusion cultivation in shake flaks, and (**b**) specific cell growth rates in logarithmic representation for GRO-I medium (■) and CDM4Avian medium (▲)

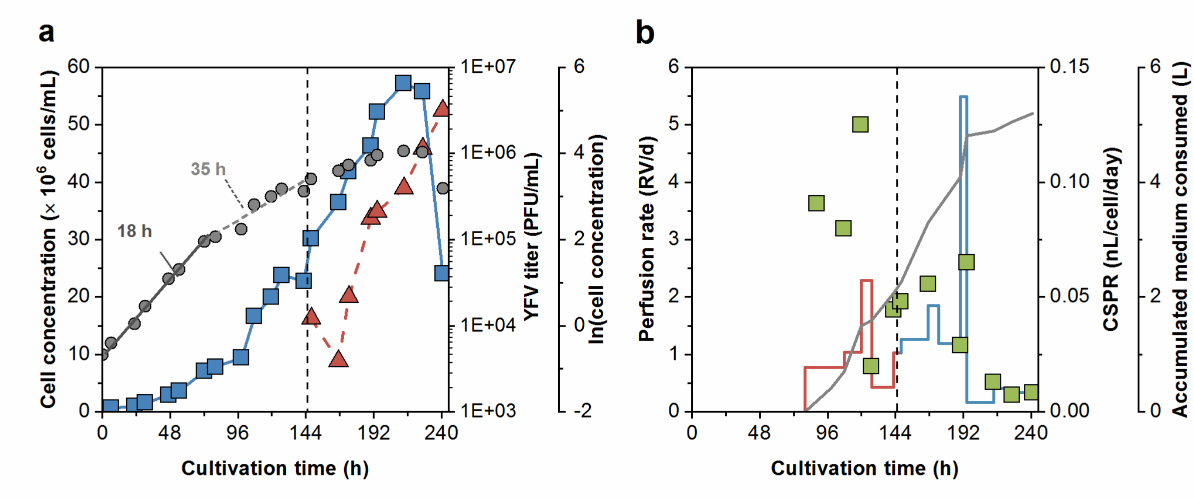

Fig. S2 TFF perfusion bioreactor cultivation of EB66® cells in GRO-I medium and infection with non-adapted yellow fever virus (YFV). (**a**) Cell concentration (■), YFV titer (▲), and the logarithmic cell growth (●). (**b**) Perfusion medium A (red line), perfusion medium B (blue line), cell-specific perfusion rates (CSPR) (■), and total volume of perfused medium (grey line). Dotted vertical line indicates time point of infection

Table S1 Primers and probes used for real-time RT–qPCR

Target	Name	Sequence (5' → 3')	Source
YFV 5' UTR	YFV_F<br>YFV_R<br>YFV_P	GCTAATTGAGGTGCATTGGTCTGC<br>CTGCTAATCGCTCAACGAACG<br>6-FAM-ATCGAGTTGCTAGGCAATAAACAC-TAMRA	Domingo et al. (2012)
ZIKV E protein	ZIKV_F<br>ZIKV_R<br>ZIKV_P	CCGCTGCCCAACACAAG<br>CCACTAACGTTCTTTTGCAGACAT<br>6-FAM-AGCCTACCTTGACAAGCAGTCAGACACTCAA-BHQ-1	Following Lanciotti et al. (2008)

References

Domingo C, Patel P, Yillah J, Weidmann M, Mendez JA, Nakoune ER, Niedrig M (2012) Advanced yellow fever virus genome detection in point-of-care facilities and reference laboratories. Journal of clinical microbiology 50(12):4054-60 doi:10.1128/jcm.01799-12

Lanciotti RS, Kosoy OL, Laven JJ, Velez JO, Lambert AJ, Johnson AJ, Stanfield SM, Duffy MR (2008) Genetic and serologic properties of Zika virus associated with an epidemic, Yap State, Micronesia, 2007. Emerging infectious diseases 14(8):1232-9 doi:10.3201/eid1408.080287
